# The hypoxia-inducible genes VEGF and *CA9* are differentially regulated in superficial *vs* invasive bladder cancer

**DOI:** 10.1038/sj.bjc.6600215

**Published:** 2002-04-22

**Authors:** K J Turner, J P Crew, C C Wykoff, P H Watson, R Poulsom, J Pastorek, P J Ratcliffe, D Cranston, A L Harris

**Affiliations:** ICRF Molecular Oncology Laboratory and Angiogenesis Group, Institute of Molecular Medicine, John Radcliffe Hospital, Oxford OX3 9DU, UK; Department of Urology, Churchill Hospital, Oxford OX3 7LJ, UK; Department of Pathology, University of Manitoba, D212-770 Bannatyne Ave, Winnipeg, Manitoba R3E 0W3, Canada; ICRF In Situ Hybridisation Service, 44 Lincoln's Inn Fields, London WC2A 3PX, UK; Institute of Virology, Slovak Academy of Sciences, 84246 Bratislava, Slovak Republic; Wellcome Trust Centre for Human Genetics, Oxford OX3 7BN, UK

**Keywords:** bladder neoplasms, angiogenic factors, carbonic acid, cell hypoxia

## Abstract

Regulation by hypoxia may underlie the expression of vascular endothelial growth factor in bladder cancer. We have compared the distribution of vascular endothelial growth factor mRNA with a hypoxia marker, carbonic anhydrase 9 (CA IX). vascular endothelial growth factor mRNA was analysed by *in situ* hybridisation and CA IX by immunochemistry in 22 cases of bladder cancer. The relationship of microvessels to the distribution of CA IX was determined. In a separate series of 49 superficial tumours, CA IX immunostaining was compared with clinico-pathological outcome. In superficial and invasive disease there was overlap in the expression of vascular endothelial growth factor and CA IX, CA IX being more widespread. Both were expressed predominantly on the luminal surface, and surrounding areas of necrosis (invasive tumours). Expression of both factors was greater in superficial disease. Expression was absent within ∼80 μm of microvessels. Unlike vascular endothelial growth factor, CA IX did not predict outcome in superficial disease. Differential responses to reoxygenation provide one explanation: vascular endothelial growth factor mRNA declined rapidly, while CA IX expression was sustained for >72 h. Expression of vascular endothelial growth factor mRNA in bladder tumours is consistent with hypoxic regulation and suggests differential regulation in superficial *vs* invasive disease. The expression of CA IX on the luminal surface justifies investigation of its utility as a therapeutic target/prognostic indicator.

*British Journal of Cancer* (2002) **86**, 1276–1282. DOI: 10.1038/sj/bjc/6600215 www.bjcancer.com

© 2002 Cancer Research UK

## 

Tumour growth and metastasis are dependent upon angiogenesis (Folkman, 1990). VEGF (vascular endothelial growth factor) is a key regulator of this process (Ferrara and Davis-Smyth, 1997). Expression of VEGF is an indicator of stage and outcome in a number of tumour types and functional studies have confirmed its central role in angiogenesis and tumour growth (Zhang *et al*, 1995; Borgstrom *et al*, 1998). Previously we demonstrated that expression of VEGF mRNA varies widely in human superficial bladder cancer and that the level of expression is predictive of relapse and stage progression (Crew *et al*, 1997). The mechanisms by which some superficial tumours express VEGF mRNA at low levels and others considerably higher (up to 345-fold more) remain undetermined. An understanding of these mechanisms may shed light on factors influencing a more angiogenic and therefore more aggressive phenotype, and could provide new therapeutic or prognostic approaches. Interestingly there are differences between superficial and invasive tumours in this regard. For example, expression of the angiogenic factor thymidine phosphorylase (TP) is significantly higher in invasive than in superficial bladder tumours (O'Brien *et al*, 1995). In contrast, expression of VEGF mRNA is four-fold higher in superficial than in invasive tumours though expression of VEGF protein does not differ (O'Brien *et al*, 1995). This is indicative of differential regulation at the translational level and we have shown that expression of the eukaryotic initiation factor-4E (eIF-4E) correlates with VEGF protein : mRNA ratios in bladder tumours (Crew *et al*, 2000).

The regulation of VEGF by hypoxia has received considerable attention. Tumour hypoxia is associated with rapid proliferation, increased risk of metastasis and poor outcome (Brizel *et al*, 1996). Regulation of gene expression by hypoxia may explain these effects. Genes regulated by hypoxia include those that facilitate anaerobic metabolism of glucose (Firth *et al*, 1994; Ebert *et al*, 1996), and genes that enhance vascularity and oxygen delivery such as VEGF (Schweiki *et al*, 1992). The transcription factor complexes HIF-1 (hypoxia inducible factor 1) and HIF-2 have emerged as key mediators of the hypoxic upregulation of these genes (Semenza, 1999).

Hypoxia is usually associated with acidic pH which may promote tumour growth (Martinez-Zaguilan *et al*, 1996). Carbonic anhydrases are important for regulation of pH and we demonstrated recently that the tumour associated carbonic anhydrase 9 (*CA9*) is tightly regulated by the HIF-1 pathway (Wykoff *et al*, 2000). (*CA9* refers to the carbonic anhydrase 9 gene including any genomic sequence and mRNA, CA IX refers to the corresponding protein). CA9 is induced strongly by hypoxia in a number of tumour cell lines and the *CA9* gene has a HIF-1 hypoxia response element (HRE) immediately 5′ to its transcriptional start site. Expression of CA IX was compared with that of the bioreductive hypoxia marker pimonidazole in a series of bladder transitional cell carcinomas. The distribution of the two factors was strikingly similar although staining for CA IX was less extensive. Both CA IX and pimonidazole were detected at the luminal surface of papillary tumours and around areas of necrosis.

Because pathways of both pH regulation (*CA9*) and angiogenesis (VEGF) are regulated by hypoxia, we have compared the expression patterns of CA IX and VEGF in superficial and invasive bladder cancers. Expression of VEGF was evaluated at the level of mRNA for two reasons. First, VEGF protein is secreted and therefore VEGF mRNA is a superior marker of the localisation of VEGF production and regulation. Second, knowledge of the localisation of the mRNA might facilitate understanding of the mechanisms of VEGF mRNA upregulation in bladder cancer, which have not been studied previously.

We demonstrate that VEGF mRNA is expressed most strongly on the luminal surface of bladder tumours, and surrounding areas of necrosis. This expression co-localises with areas of tumour hypoxia as defined by expression of CA IX in both superficial and invasive bladder tumours. We also show that expression of CA IX is greater in superficial than invasive tumours, consistent with previous observations on the expression of VEGF mRNA (O'Brien *et al*, 1995). These observations highlight differences between superficial and invasive bladder cancer, suggesting that different pathways may regulate angiogenesis in superficial and invasive disease with the possibility that hypoxia is the driver of angiogenesis in papillary tumours.

## MATERIALS AND METHODS

### Immunohistochemistry

Formalin-fixed, paraffin-embedded tissue specimens collected by standard surgical oncology procedures were obtained from the Pathology Department, John Radcliffe Hospital, Oxford, UK. Samples of normal bladder were taken from cadaveric organ donors at the time of nephroureterectomy. CA IX immunostaining using monoclonal antibody M75 was as described (Wykoff *et al*, 2000). Slides were viewed by two observers (K Turner and P Watson). The CA IX score was derived from the product of (i) the percentage of tumour cells staining for CA IX and (ii) the average intensity of that staining on a scale of 1 (least intense) to 3. M75 antibody was from Pastorek (Pastorek *et al*, 1994).

### Double staining for CA IX and CD34

CA IX immunostaining was as described with omission of counterstaining (Wykoff *et al*, 2000). Sections were then incubated with 1 : 100 MoAb QBEnd10 (DAKO) (30 min) and then with goat anti-mouse IgG (PO447, DAKO) (30 min) followed by APAAP (30 min). The last two steps were repeated twice with 10 min incubations. Visualisation was by New Fuchsin Red substrate (DAKO). The distance from vessels to the edge of regions of CA IX staining was assessed using an eyepiece graticule calibrated against a graduated slide. Vessels that lay obliquely to the plain of the section were excluded.

### *In situ* mRNA hybridisation

Specific localisation of VEGF mRNA was accomplished by *in situ* hybridisation using an antisense riboprobe as described (Wykoff *et al*, 2000).

CA IX and VEGF mRNA were studied in serial tissue sections. In the majority of cases CA IX expression was more widespread than expression of VEGF mRNA. For this reason, the percentage of the tumour expressing VEGF mRNA that was also positive for CA IX was quantified in each section. Sections were viewed by two observers (K Turner and P Watson) and a consensus reached.

### Effect of reoxygenation on hypoxically induced CA IX protein/VEGF mRNA tissue culture

The A549 lung carcinoma cell line was obtained from ECACC (European Collection of Animal Cell Cultures, UK). Cells were grown in DMEM (Sigma) supplemented with 10% foetal calf serum (Globepharm), L-glutamine (2 μM), penicillin (50 IU ml^−1^), and streptomycin sulphate (50 μg ml^−1^). Studies of gene expression were performed on cells approaching confluence in normal growth medium. Parallel incubations were performed on aliquots of cells in normoxia (humidified air with 5% CO_2_) and hypoxia. Hypoxic conditions were generated in a Napco 7001 incubator (Precision Scientific) with 0.1% O_2_, 5% CO_2_, and balance N_2_. Re-oxygenation experiments were performed by exposure of cells to hypoxia for 16 h followed by a return to normoxia for the indicated time.

### RNA analysis

Total RNA was extracted by a modified acid/guanidinium thiocyanate/phenol/chloroform method (RNAzol B; Cinna/Biotec Laboratories), dissolved in hybridisation buffer (80% formamide, 40 mM PIPES, 400 mM sodium chloride, and 1 mM EDTA, pH 8) and analysed by RNase protection assay (RPA). RPAs for vascular endothelial growth factor-A (VEGF-A) and *CA9* were performed using ^32^P-labelled RNA probes transcribed using SP6 RNA polymerase from the previously described DNA templates (Maxwell *et al*, 1999; Wykoff *et al*, 2000). RPAs were performed on 30 μg total RNA using an internal control assay for U6 small nuclear RNA as described (Maxwell *et al*, 1999).

### Cell lysis and immunoblotting

Whole cell protein extracts were prepared from tissue culture cells by 10 s homogenisation in denaturing conditions as described (Wiesener *et al*, 1998). For Western analysis, aliquots were separated by SDS-polyacrylamide gel electrophoresis and transferred to Immobilon-P membranes. CA IX was detected using the mouse monoclonal anti-human CA IX antibody M75 (1 : 50) as described (Pastorekova *et al*, 1992). HRP-conjugated goat-anti-mouse immunoglobulin (DAKO) (1 : 2000) was applied for 1 h at room temperature (RT). ECL Plus (Amersham Pharmacia) was used for visualisation.

## RESULTS

### Expression of VEGF mRNA in bladder cancer

Expression of VEGF mRNA was examined by *in situ* hybridisation in 22 cases of bladder cancer selected to represent the range of stage and grade in human bladder tumours (Table 1

**Table 1:**
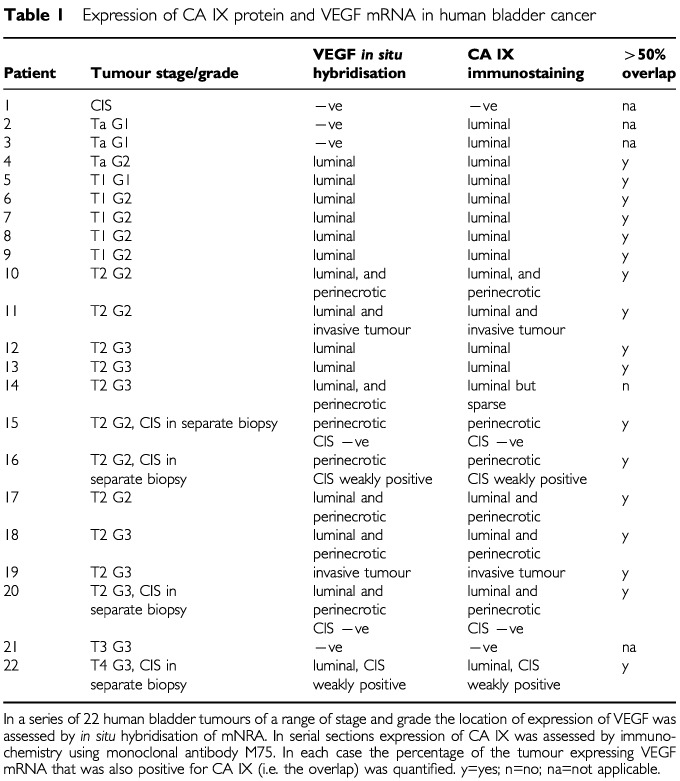
Expression of CA IX protein and VEGF mRNA in human bladder cancer

), and in two samples of normal bladder. Expression of a control mRNA (β-actin) was verified in each case as a marker of mRNA preservation (data not shown). Neither of the samples of normal bladder showed any expression of VEGF mRNA (data not shown). There was considerable inter-tumour variation in VEGF with no expression detected in four cases (one case of isolated carcinoma *in situ* (CIS), two TaG1 tumours, and one T3G3 tumour). In the remaining cases expression was most intense on the luminal surface of tumours. This expression was patchy, with areas of strong and weak luminal expression within the same resected chip. Enhanced luminal expression was particularly marked in superficial tumours: intense staining of the outer half of the transitional cell epithelium was typical (approximately 50 μm from luminal surface to core) (Figure 1

**Figure 1:**
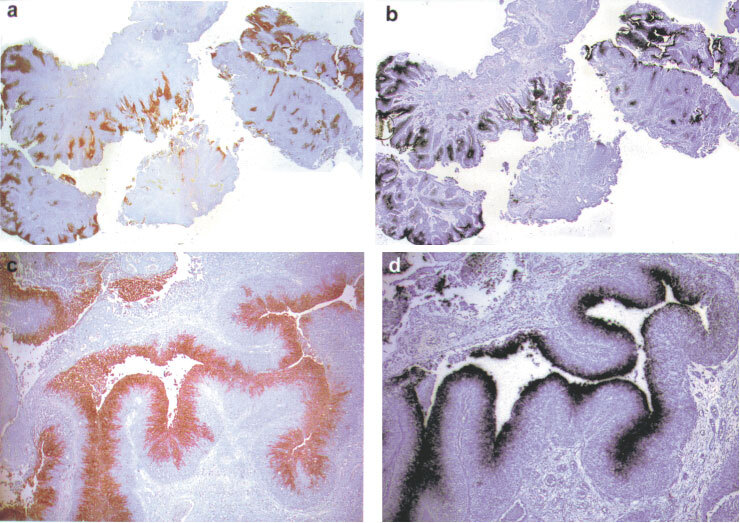
Expression of CA IX protein (**A**,**C**) and VEGF mRNA (**B**,**D**) in superficial bladder cancer at low power (reduced from×10) (**A**,**B**) and high power (reduced from×40) (**C**,**D**).

). Increased luminal expression was also noted in all chips of invasive tumour that contained surface epithelium. However, this staining was substantially less intense and widespread than in superficial tumours (Figure 2

**Figure 2:**
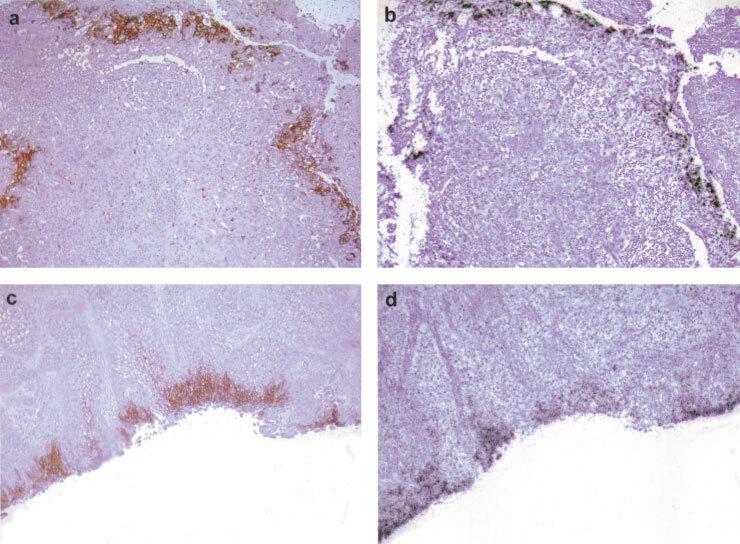
Expression of CA IX protein (**A**,**C**) and VEGF mRNA (**B**,**D**) in invasive bladder cancer showing perinecrotic (**A**,**B**) and luminal expression (**C**,**D**). Reduced from×40.

). In several cases of invasive tumour, in addition to luminal expression, there was enhanced expression of VEGF within the invasive portion of the tumour. This expression was observed primarily around areas of necrosis (Figure 2). Of the four cases of invasive bladder cancer in which there was CIS in a separate biopsy, the CIS did not express VEGF in two cases and was weakly positive in the remaining two cases.

### Expression of CA IX

CA IX expression was evaluated by immunochemistry in the same 22 cases on serial sections. In 17 out of 18 cases in which VEGF mRNA was detected, at least 50% of the areas that expressed VEGF mRNA also expressed CA IX. Furthermore, the distribution of CA IX and VEGF expression was strikingly similar (Figures 1 and 2). Like VEGF mRNA, CA IX was expressed maximally on the luminal surface of tumours and around regions of necrosis in invasive tumours. In addition, whilst both superficial and invasive tumours showed luminal enhancement, this was much more marked in superficial tumours. Expression of CA IX in the sections of CIS from patients with invasive tumours matched that of VEGF. CA IX was not detected in the normal bladder specimens, nor was it detected in samples from the patient with isolated CIS.

In addition to these 22 cases, CA IX immunostaining was performed on a further five cases of isolated CIS, three normal bladders and four normal ureters. Of these, one case of CIS showed very weak luminal staining, but the remainder of the CIS cases and all sections of normal urothelium were negative for CA IX expression. In summary, in a total of 10 cases of CIS, immunostaining for CA IX was weak in three, and absent in seven.

### Relationship of CA IX to microvessels

**Figure 3:**
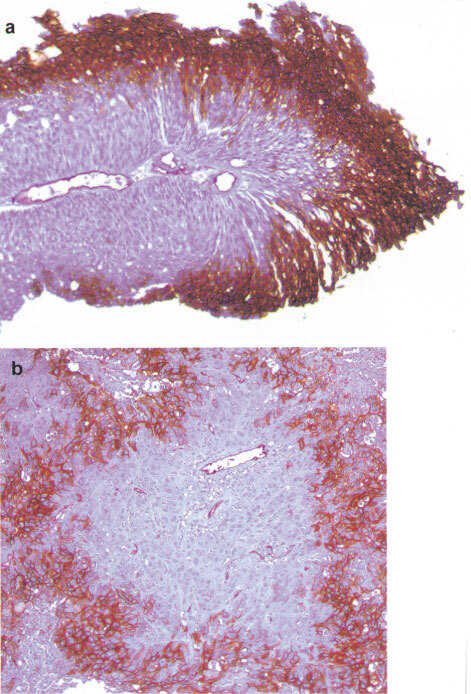
Relationship of CA IX expression to microvessels in superficial (**A**) and invasive bladder cancer (**B**). Reduced from×40.

In order to elucidate factors that might influence the expression of CA IX in bladder cancer, we examined the relationship between expression of CA IX and tumour microvessels. The distribution of CD 34 staining (as a marker of blood vessels) was compared to that of CA IX staining in the same sections for a subset of cases (52 vessels were assessed in 12 different cases, Figure 3

). In superficial and invasive tumours, CA IX expression was typically detected in regions of the tumour that were a mean distance of 80 μm from a blood vessel (standard deviation=44 μm).

### Relationship of CA IX expression in superficial bladder cancer to other clinical and prognostic factors

We have demonstrated previously that VEGF mRNA expression on RNAse protection was predictive of recurrence and stage progression in a series of 55 superficial bladder tumours (Ta/T1, G1/G2) (Crew *et al*, 1997). Given the association between CA IX and VEGF expression, we aimed to establish whether CA IX expression was also predictive of outcome in these cases. Therefore, sections from 49 of these previously studied cases were stained for CA IX and expression was scored semi-quantitatively (range=0–225, median=30, cases were split into ‘low’ CA IX (i.e. score <median (*n*=22), and ‘high’ CA IX (i.e. score >median (*n*=27)). Comparisons were made between CA IX expression and other factors that we had determined previously including expression of VEGF mRNA (by RNAse protection), tumour grade, risk of recurrence, time to recurrence, and risk of stage progression (Table 2

**Table 2:**
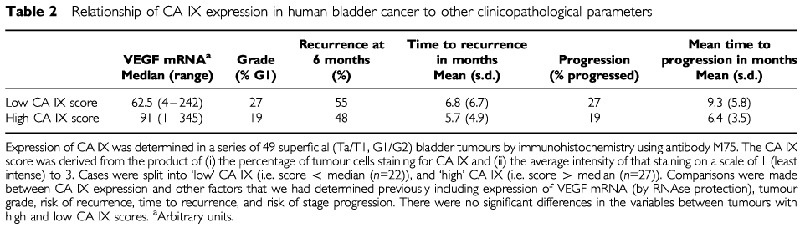
Relationship of CA IX expression in human bladder cancer to other clinicopathological parameters

). Although we had observed concordance of the focal staining pattern in superficial tumours, the total level of VEGF mRNA as assessed by RNAse protection did not correlate statistically with the CA IX score in these cases. No significant associations were found between CA IX staining and any other of these parameters when assessed by both continuous and cutpoint analysis.

### Temporal relationship of CA IX/VEGF expression to hypoxia and reoxygenation

**Figure 4:**
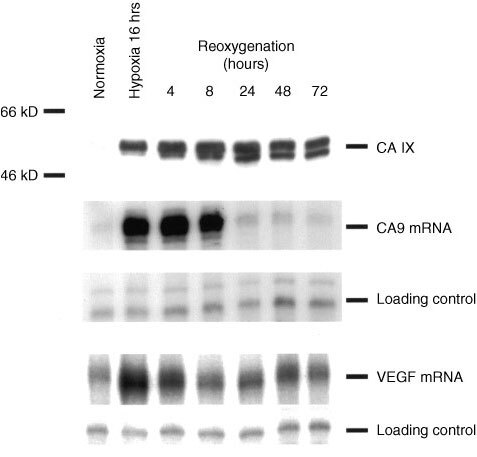
Expression of CA IX protein (by Western blotting), and VEGF mRNA/*CA9* mRNA (by RNAse protection) in A549 lung carcinoma cells after growth under hypoxic conditions and then subsequent reoxygenation.

Though there was striking overlap in the expression of CA IX and VEGF, there were differences in the extent of the expression of these two factors. Specifically, CA IX was generally expressed over a greater area of the tumour than VEGF mRNA, and VEGF but not CA IX was predictive of time to recurrence and risk of stage progression in Ta/T1 disease. We therefore investigated any differences in regulation that might underlie these observations. In particular we investigated the temporal relationship between expression of CA IX/VEGF and hypoxia and reoxygenation. To our knowledge, there are no currently available cell lines derived from superficial human bladder tumours. Previously we had been unable to detect hypoxic induction of CA IX in invasive bladder cancer cell lines (Wykoff *et al*, 2000), which is consistent with the low level of CA IX expression observed in invasive tumours in this study. For these reasons the A549 lung carcinoma cell line, which shows marked hypoxic induction of both VEGF and *CA9*, was chosen for these studies. Interestingly, whilst *CA9* mRNA and CA IX protein, and VEGF mRNA were induced by hypoxia, they differed markedly in their response to reoxygenation (Figure 4

). Detection of CA IX protein remained constant for at least 72 h but both *CA9* mRNA and VEGF mRNA declined rapidly, reaching normoxic levels of expression within 8 h.

## DISCUSSION

Angiogenesis plays a key role in the pathogenesis of bladder cancer: microvessel density is a prognostic indicator in invasive bladder cancer (Bochner *et al*, 1995; Jaeger *et al*, 1995), expression of the angiogenic factors bFGF (basic fibroblast growth factor), TGFβ (transforming growth factor β), midkine, and HGF (hepatocyte growth factor) is elevated in patients with bladder cancer (reviewed in Jones and Fujiyama, 1999) and VEGF expression is predictive of stage progression and relapse in superficial tumours (Crew *et al*, 1997).

Current attention is focused on the upstream events that govern expression of angiogenic mediators. Hypoxia has emerged as a potent stimulus in this regard, with hypoxia inducible transcription factors regulating many aspects of this hypoxic response, including expression of VEGF. The activity of the HIF pathway can also be modulated by genetic influences such as mutations in the VHL gene and other oncogenes (Maxwell *et al*, 1999), and by growth factors (Semenza, 2000).

In this study we have investigated the relationship between expression of VEGF and CA IX, having shown previously that *CA 9* is upregulated by HIF. We demonstrate that expression of VEGF mRNA localises to areas of the tumour that also express CA IX in both superficial and invasive bladder cancers. These observations suggest that expression of VEGF in human bladder cancer is determined by tumour hypoxia and may be mediated via the HIF pathway.

Expression of both VEGF and CA IX was most intense on the luminal surface of tumours. Malignant transitional epithelium is many times thicker than transitional cell epithelium in normal bladder. The reduced oxygen tension that is associated with this increased distance from vessels may enhance expression of VEGF.

Increasingly, superficial and invasive bladder cancers are regarded as distinct pathological entities and a two-pathway model for bladder tumour development has been proposed (see Lee and Droller, 2000 for review). In keeping with this, superficial and invasive bladder tumours differ in their expression of angiogenic mediators. For example, expression of VEGF is four-fold higher in superficial tumours than in invasive tumours but expression of thymidine phosphorylase is 33 times higher in invasive tumours (O'Brien *et al*, 1995). In concordance with these results, we show here that expression of VEGF mRNA is more widespread in superficial tumours than in invasive tumours.

The localisation, shown here for the first time, of VEGF mRNA on the luminal surface of bladder tumours explains the strong relationship we have found previously between urinary and tumour VEGF protein, and the relationship of urinary VEGF to recurrence (Crew *et al*, 1999). Expression of CA IX/VEGF mRNA at the luminal surface of invasive tumours was markedly less than that on the luminal surface of superficial tumours, even in regions of equivalent distance from vessels. In addition, expression of CA IX/VEGF mRNA was observed around areas of necrosis in invasive tumours, a phenomenon that we have observed in tumours of the breast, ovary, and head and neck (Wykoff *et al*, 2000). This perinecrotic expression of CA IX/VEGF was also markedly less intense than that observed on the luminal surface of superficial tumours, even though microenvironmental hypoxia is the likely precipitant. These data therefore suggest that there is relatively less activation of hypoxia regulated transcription pathways in invasive tumours. This apparent difference between the two tumour types adds to the evidence that superficial and invasive bladder cancer represent different disease processes.

Support for this hypothesis comes from our observations in CIS, which is currently regarded as a progenitor of the invasive rather than superficial phenotype (Lee and Droller, 2000). Like invasive tumours, expression of VEGF/CA IX in CIS was substantially less than that seen in superficial bladder cancer, even though the transitional epithelium in CIS was equivalent in thickness to superficial bladder cancer.

Expression of CA IX was generally more widespread than that of VEGF mRNA, and VEGF but not CA IX was predictive of relapse and stage progression in our series of superficial bladder tumours. There are several potential explanations for this discrepancy. First, the difference in distribution may reflect different temporal responses to cellular hypoxia. Whilst both factors were strikingly induced by 16 h of hypoxia in tissue-culture cells, the level of VEGF mRNA declined rapidly upon reoxygenation (in accordance with a previous description (Schweiki *et al*, 1992)) whereas CA IX protein levels remained high during reoxygenation for at least 72 h. Therefore, the expression of VEGF mRNA observed in bladder tumours may reflect current hypoxia, whereas expression of CA IX may represent more chronic hypoxia. Second, whilst expression of CA IX may facilitate survival under adverse pH, expression of VEGF enhances growth and implantation. Hypoxia stimulates the expression of both factors, but the level of expression of each factor will vary and the factor that most favours tumour growth or implantation is most likely to correlate with adverse outcome. Third, in addition to regulation by HIF, *CA9* and VEGF most likely have other dissimilar mechanisms of regulation. For instance, VEGF expression in hypoxia is also influenced by the RNA binding protein HuR that stabilises VEGF mRNA (Levy *et al*, 1998).

This study emphasises the importance of hypoxia in defining the patterns of gene expression in bladder cancer. It also adds to the growing body of evidence that superficial and invasive bladder cancer exhibit significant biological differences. In particular, the former demonstrate a much more marked luminal expression of hypoxia inducible genes. Expression of extracellular carbonic anhydrases is likely to affect microenvironmental pH and in doing so may promote tumour growth (Martinez-Zaguilan *et al*, 1996). Carbonic anhydrase inhibitors inhibit the invasion of renal cancer cells *in vitro* and synergise with chemotherapeutic agents in animal models (Teicher *et al*, 1993; Parkkila *et al*, 2000). The high frequency and marked enhancement of CA IX expression in superficial bladder cancer reported here, combined with the relative absence in normal transitional epithelium, suggests that investigation into the utility of CA IX as a therapeutic target in this context is warranted. Furthermore, since CA IX is a transmembrane protein, measurement of shed protein in the urine could be a potential marker of recurrence.
